# Pathophysiology of Vaccine-Induced Prothrombotic Immune Thrombocytopenia (VIPIT) and Vaccine-Induced Thrombocytopenic Thrombosis (VITT) and Their Diagnostic Approach in Emergency

**DOI:** 10.3390/medicina57100997

**Published:** 2021-09-22

**Authors:** Pierpaolo Di Micco, Giuseppe Camporese, Giuseppe Cardillo, Corrado Lodigiani, Novella Carannante, Anna Annunziata, Giuseppe Fiorentino, Vincenzo Russo, Egidio Imbalzano

**Affiliations:** 1 UOC Medicina, Ospedale Buon Consiglio Fatebenefratelli, 80122 Napoli, Italy; 2 Unit of Angiology, Azienda Ospedaliera di Padova, 35100 Padova, Italy; giuseppe.camporese@aopd.veneto.it; 3 Medylab, Lusciano, 80100 Napoli, Italy; giuseppe.cardillo.75@gmail.com; 4 Thrombosis and Haemorragic Diseases Unit, IRCCS Humanitas Research Hospital, 20089 Milan, Italy; corrado.lodigiani@humanitas.it; 5 Emergency Unit, Ospedale D. Cotugno, 80131 Napoli, Italy; carannantenovella@gmail.com; 6 Department of Cardiology, OspedaleMonaldi, 80131 Napoli, Italy; annunziata.anna@gmail.com (A.A.); giuseppefiorentino1@gmail.com (G.F.); 7 Head of Cardiology, University of Campania “Luigi Vanvitelli”, 80100 Napoli, Italy; v.p.russo@libero.it; 8 Department of Cardiology, University of Messina, 98100 Messina, Italy; egidio.imbalzano@unime.it

**Keywords:** SARS-CoV-2, COVID-19, anti-SARS-CoV-2 vaccine, vaccine, pandemic

## Abstract

SARS-CoV-2 induced a pandemic that is reported to have started in Asia and was then extended to other countries in the world. Main clinical aspects of this viral infection have been lung injuries with severe pneumonia requiring prolonged hospitalization and associated morbidities such as venous thromboembolism and/or superinfection by bacteria, fungus or other pests. Immediately there was a need to develop a sustainable therapeutic strategy, such as vaccination. Vaccines against Covid-19, in fact, exert a protective action for common people and reduce viral diffusion. Yet, vaccination of a large number of people raises the question of a well-known complication of several types of vaccines; this complication is immune thrombocytopenia, which is sometimes associated with thrombosis as well. In this short review, we summarized mechanisms involved in the pathogenesis of vaccine-induced prothrombotic immune thrombocytopenia and vaccine-induced thrombocytopenic thrombosis.

## 1. Background

Since the SARS-CoV-2 pandemic began, several strategies to reduce diffusion of contagion have been adopted around the world. For this reason, after understanding the mode and details of transmission of SARS-CoV-2, social distancing, protective masks, gloves and several other restrictive measures have been adopted worldwide in order to prevent the spread of COVID-19, awaiting the subsequent vaccination campaign [1]. The time taken to obtain the first vaccine against SARS-CoV-2 has been short; this is historically relevant, guaranteeing in the shortest time the attempt for early cessation of the pandemic [2]. For this reason, since the vaccination campaign began, great attention has been reserved for unexpected side effects of each type of anti-SARS-CoV-2 vaccine [3,4]. In this field, great attention has been reserved for vaccine-induced prothrombotic immune thrombocytopenia (VIPIT) and for vaccine-induced thrombotic thrombocytopenia (VITT) because they may be associated with poor outcomes. VIPIT has been reported as a complication of every type of anti-SARS-CoV-2 vaccine (e.g., Pfizer, Moderna, AstraZeneca and Johnson & Johnson); sometimes this feature is also associated with bleeding and thrombotic complications (i.e., VITT) [5,6]. Yet, the underling pathophysiological mechanism may be considered to be similar to other causes, in particular, other types of vaccines [7]. In the following paragraphs, we report pathogenetic mechanisms inducing immune thrombocytopenia with thrombotic microangiopathy after vaccination against SARS-CoV-2 as well as after vaccination against other viruses, and we will also describe mechanisms able to induce other types of thrombocytopenia/thrombocytopathy. In the final paragraph, we suggest a model to manage the differential diagnosis of VIPIT/VITT. In order to accomplish this, a thorough analysis of the literature was performed.

## 2. Search Methods

For this update, MEDLINE, EMBASE and PubMed were searched up to 24 June 2021 for studies that evaluated the incidence of VITT after vaccination. VIPIT, VITT, anti-SARS-CoV-2 vaccine, autoimmune thrombocytopenia, immunological thrombocytopenia and anti-COVID-19 vaccine have been used as terms for this research.

## 3. Immune Prothrombotic Thrombocytopenia after Antiviral Vaccines

Vaccines are the most effective treatment for prevention of infections, as demonstrated for poliomyelitis, smallpox and other infections [8].

Despite their scientific relevance, vaccines are often the subject of debate due to adverse events. For this reason, health authorities usually recommend the collection of vaccine-related adverse events in any case.

Thrombocytopenia is an adverse event that has been associated with several types of vaccines as well as with viral infections [9,10,11]. It has been estimated that the underlying pathophysiological mechanism is immunity. Antibodies against platelet factor 4 (PF4) can be detected on platelets of subjects that develop thrombocytopenia after vaccines in about 79% of cases [12]; thus, it may be considered as immune thrombocytopenia. However, being a clinical problem described after several vaccines, proper clinical surveillance of immune thrombocytopenia is difficult, particularly in terms of frequency and severity. 

It has been suggested that the vaccination more frequently associated with immune thrombocytopenia is measles-mumps-rubella (MMR) [12], although post-MMR-vaccine immune thrombocytopenia is significantly less common than after each one of the three clinical infections.MMR vaccine may induce immune thrombocytopenia with an incidence of 1 in 25,000–40,000 vaccinations; spontaneously or after treatment, restoration of platelet numbers is usually observed in 2–3 weeks, while less than 10% of patients develop chronic thrombocytopenia [7,12,13]. Furthermore, immune thrombocytopenia has also been detected after vaccines against tetanus and poliomyelitis [12].

The clinical course of immune thrombocytopenia is usually not complicated and is not associated with bleeding or thrombosis; for this reason, VIPIT has never been considered a limitation for the use of vaccines in children [12,13].

Regarding the pandemic of SARS-CoV-2, we know that in the last two years, the spread due to SARS-CoV-2 began and the world has been upset. So, at the end of 2020 and the beginning of 2021, several vaccines have been tested and then licensed from the European Medicines Agency (EMA) to fight the pandemic diffusion of SARS-CoV-2: mRNA-based vaccines—BNT162b2 (Pfizer-BioNTech, Reinbek, Germany) and mRNA-1273 (Moderna, Cambridge, MA, USA), as well as recombinant vector vaccines, ChAdOx1 nCov19 (AstraZeneca, Cambridge, UK) and Ad26.COV2.S (Johnson & Johnson/Janssen, New Brunswick, NJ, USA), have been the most commonly used in Europe and around the world.

Safety concerns have been raised by the European Union because of the occurrence of specific micro-thromboses associated with thrombocytopenia after the first dose of vaccine, especially after the first dose of attenuated virus vaccine, as described in the literature [14,15,16]. The localization of those thromboses is usually in cerebral vessels and the clinical course may be influenced by neurological complications, such as cerebral hemorrhage. Along with clinical symptoms such as headache, visual changes and/or focal neurological signs, an increase of d-dimer or other inflammatory markers has also been reported after the anti-SARS-CoV-2 vaccine [17] and this is related to the activation of the cytokines network. 

Intriguingly, pathophysiology of these thrombotic processes may induce in the same subject thrombosis and hemorrhages: bleeding is due to the reduced number of platelets and the platelet lysis may also induce a prothrombotic state because of the release of PF4 that acts as heparin binding factor [18,19].

mRNA anti-SARS-CoV-2 vaccines seem to be associated mainly with the occurrence of VIPIT and rarely with VITT with neurological damages [20,21,22], while, as mentioned above, inactivated whole-virus anti-SARS-CoV-2 vaccines seem to be more frequently associated with intracranial VITT, with the occurrence of immune thrombocytopenia and cerebral venous thrombosis [14,15,16,17]. Because of the association with the prothrombotic state, immunopathological thrombocytopenia and clinical neurological complications after anti-SARS-CoV-2 vaccine, EMA confirmed the acronym of VITT, “vaccine-induced thrombotic thrombocytopenia”, in which there is massive platelet activation, low platelet count and blood clots, with diffuse venous thrombosis, in particular in atypical sites such as the brain and rarely the abdomen. First symptoms, such as fatigue, abdominal discomfort and headache, appear between the 5th and the 28th day after the first dose of anti-SARS-CoV-2 vaccine in healthy patients; most cases occur in women under 60 years old [14,15,16,17,18,19,20,21].Furthermore, because of the need to perform vaccines all around the world as soon as possible in order to eradicate the SARS-CoV-2-pandemic, VIPIT and VITT arecapturing the attention of individuals and the media; fear, in fact, is influencing public opinion, although the incidence of VIPIT/VITT is not high.

## 4. Thrombocytopenia and Thrombocytopathy Due to Other Causes

Pathophysiological processes reported for heparin-induced thrombocytopenia with thrombosis (HITT) have been raised as also being suggestive for VITT. This syndrome is similar to “heparin-induced thrombocytopenia” (HIT), in which heparin is the trigger for the development of disease. Pathophysiologically, unfractionated heparin (UFH) or low-molecular-weight heparin (LMWH) reacts with platelet factor 4 (PF4), producing immunogenic complexes, recognized by specific autoantibodies that lead to HIT between the 5th and 10th day after drug administration, with very high risk of thrombosis [22,23,24]. From a diagnostic point of view, several laboratories have shown that these antibodies recognize a complex of heparin with platelet factor 4 and so the dosage of PF4-heparin complexes has been confirmed in those cases as the golden standard to confirm this diagnosis [24]. The clinical scenario of HITT is dominated by thrombotic complications although the platelet count may be very low [25,26].

As previously reported, the mechanism of VITT has been clearly explained because the immune thrombocytopenia and related thrombocytolysis promote thrombus formation in a similar way for HITT; antibodies against PF4-heparin complexes were identified during both diseases, thus confirming that pathophysiological mechanisms of HITT and VITT are similar. 

Yet, HITT is not the only diseases associated with thrombocytopenia with thrombotic complications; other clinical immune thrombocytopenias are also frequently associated with those clinical complications.

Immune thrombocytopenia (ITP) is an autoimmune disorder in which a combination of defective platelet production and enhanced clearance leads to thrombocytopenia. Many cells of the immune system are involved, which promotes the establishment of an integrated and complex autoimmune mechanism. The destruction of platelets is favored through T cells that stimulate B cells to produce autoantibodies against platelets and encourage their destruction. Simultaneously, a production defect by the bone marrow evolves, because of immune activity against megakaryocytes and insufficient stimulation of thrombopoietin [7,27]. The primary aim for therapy in patients with this condition is the prevention of bleeding. However, more recently, increased rates of venous and arterial thrombotic events have been reported in ITP, even in the context of marked thrombocytopenia [28].

Furthermore, antiphospholipid syndrome (APS) is one of the most common diseases in which thrombocytopenia is associated with thrombosis. Thrombocytopenia in that disease is considered as one of the diagnostic criteria used to perform the correct diagnosis of APS according to several consensuses [29,30]. From a clinical point of view, thrombocytopenia in patients with APS is mild and rarely associated with bleeding, while mechanisms that induce thrombocytopenia are still the matter under discussion, although the binding of antiphospholipid antibodies on the platelet surface, inducing platelet activation and thrombus formation, has been clearly understood [30]. APS may appear as an isolated disease or may be associated with a systemic disease such as connectivitis or erythematosus systemic lupus (ESL) [30,31,32].

Another autoimmune disease that may appear to be associated with immune thrombocytopenia, systemic autoimmunity and induced thrombosis is Evans syndrome. Usually the main clinical manifestation of Evans syndrome is hemolyticanemia that may be associated with immune thrombocytopenia and Coombs’ positive test [33,34]. Evans syndrome may also be associated with systemic autoimmune disease such as ESL [33,34]. Signs and symptoms of Evans syndrome may vary from fatigue and purpura to thrombotic microangiopathies [33]. 

Furthermore, other immune thrombocytopenias may be associated with progressive thrombocytopenia and thrombotic microangiopathy, such as thrombotic thrombocytopenic purpura (TTP) [35]. It is a rare and life-threatening thrombotic microangiopathy characterized by microangiopathic hemolytic anemia, severe thrombocytopenia and organ ischemia linked to small vessel thrombosis [36]. Immunopathological conditions such as connectivitis or bacterial sepsis or recent viral infections are the main predisposing diseases for patients that developed TTP. Moreover, in this disease, the neurological scenario is compromised, along with the frequent involvement of kidney. Microthrombosisis frequently identified on autopsy without a clear disseminated intravascular coagulation. Pathophysiological mechanisms are different and are related to immune dysfunction of ADAMS T13 protease [36,37]. Congenital defects of ADAMS T13 may be associated with similar clinical features but with an early onset of diseases in juvenile age [38].

Of course, during a differential diagnosis in patients showing thrombosis of small vessels and thrombocytopenia, drug-induced thrombocytopenia should also be evaluated. The clinical diagnosis is based mainly on anamnesis, while laboratory and instrumental findings offer strong support to exclude other clinical causes of thrombocytopenia with thrombotic complications [39,40].

## 5. Discussion

The world experienced dread regarding the occurrence of VIPIT and/or VITT after SARS-CoV-2 vaccines. Scientific reports underlined that AstraZeneca or Johnson and Johnson vaccines are more frequently associated with VITT. Pathophysiological mechanisms of VIPIT refer to a specific type of blood clot associated with immunological abnormalities after vaccination [41]. These mechanisms are different from the blood’s regular clotting mechanism or conditions like deep vein thrombosis, while seeming to be similar to those described for other types of vaccines, although further studies may be useful on this topic [42]; furthermore, as far as for other viral infections, immune thrombocytopenia may be a consequence of infection by SARS-CoV-2 [43] and this condition should always be first taken into account to confirm or disconfirm a diagnosis of VITT/VIPIT. In clinical practice, the confirmation of occurrence of any other type of immune thrombocytopeniais usually performed after the determination of platelet reduction along with determination of anti-platelet antibodies, while pathophysiological known mechanisms of VIPIT are associated with the recent vaccination and the detection of anti-PF4-heparin antibodies that are able to induce thrombosis of small vessels.

For this reason, the European Medical Agency (EMA)underlined on 6 April that it had found a “possible link” between the AstraZeneca COVID-19 vaccine and the very rare blood clots [44]. Cases of VITT have been primarily reported, 4–28 days after vaccination, in women under the age of 55 (less frequently in men), in unusual venous vessels or in arterial vessels (i.e., brain vessels), in a condition in which platelet count progressively decreases, in the presence of increased d-dimer and normal or decreased fibrinogen and with positive Ig versus PF4-Hep complexes with ELISA testing (Table 1). 

**Figure 1:**
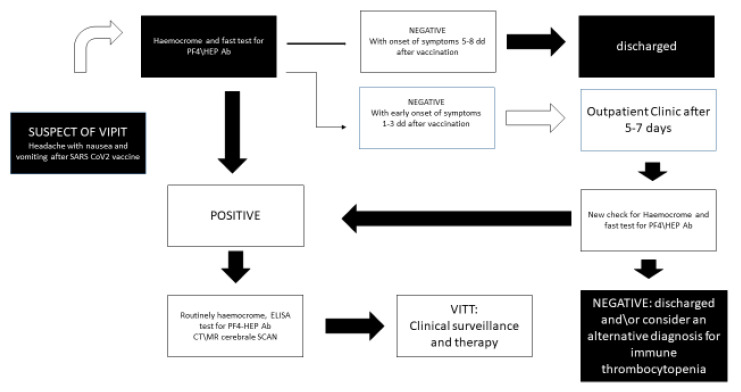
Flow chart to manage patients with suspected VIPIT\VITT at the emergency room.

Yet, because immune thrombocytopenia with associated thrombosis may also be found in other clinical conditions, as we previously reported, we suggest in the case of negativity of ELISA test to detect anti-PF4-Hep ab to consider other clinical conditions associated with immune thrombocytopenia and thrombosis (Table 2 and Figure 1). From a clinical point of view, this last information appears as fundamental in the triage management of patients with symptoms of suspected VITT because the increase of vaccinated people includes patients that potentially have thrombocytopenia due to other causes. 

The triage is a complex system in which vital signs, referred symptoms, personal anamnesis and objective clinical signs should be evaluated a few times when making a clinical surmise. Therefore, along with easily obtained data such as sex and age, during the triage system the description of headache may have clinical relevance together with the vigilance of the patient. Of course, in the case of extensive cerebral thrombosis symptoms such as headache, visual disturbances and nausea, they may be associated with vomiting, obfuscation, fatigue or pre-syncopal state and seizures. For this reason, vital signs may not be suggestive by themselves: non-extensive cerebral thromboses usually show only mild bradycardia, while extensive cerebral thromboses may show low blood pressure, moderate or severe bradycardia, bradypnea or superficial breathing. This complex clinical scenario always needs further examinations with blood samples and radiological imaging. The presence of thrombocytopenia is useful but if the anamnesis reveals a further cause of possible thrombocytopenia (see Table 2), a dosage of anti-PF4-Hep is required to improve the acquisition of data for a fast diagnosis of VITT. However, the objective confirmation of VITT may be performed only after radiological imaging with a cerebral CT/MR scan (Figure 1).

So, correct management of the clinical features should be focused on the triage system to confirm/exclude VITT/VIPIT as well as for other acute medical illnesses: anamnesis, clinical features and differential diagnosis should always be integrated with useful laboratory markers and radiological imaging. 

In the case of exclusion of VITT, acquired information may be used to better direct clinical suspicion toward other diseases associated with acute/subacute thrombocytopenia (Table 2). In this way, the flow that we reported in Figure 1 takes into account information obtained by the triage system and the clinical picture, and it uses available information from laboratory biomarkers and radiological imaging to offer a fast track for patients with suspected VITT.

## 6. Conclusions

VIPIT occurring after a vaccination may pass into an asymptomatic state or it may have severe clinical complications as VITT. The occurrence of VIPIT/VITT has been demonstrated after the administration of several anti-SARS-CoV-2 vaccines. Yet, VIPIT and VITT have also been reported after other types of vaccines, but pathophysiological mechanisms seem to be different from other immune thrombocytopenias as far as the clinical course because VITT may affect brain venous vessels. Useful laboratory markers to detect VIPIT/VITT are present but clinically, a correct diagnosis may retain several difficulties because thrombosis usually occurs in atypical sites (e.g., cerebral vein thrombosis). Most common referred neurological signs and symptoms of cerebral VITT depend on the extension of damage and the only useful information is represented by the anamnesis of recent vaccination in an era in which anti-SARS-CoV-2 vaccinations are performed on a large scale. So, fast clinical surmise and an easy flow chart that considers referred symptoms, clinical signs, differential diagnosis and laboratory and radiological examinations may be useful in performing diagnosis of VITT. This last aspect takes into account the precious fast diagnosis that is useful for all acute illness in emergency departments, in particular when this dangerous complication of anti-SARS-CoV-2 vaccination is considered.

## Figures and Tables

**Table 1:** Clinical characteristics for suspected VIPIT/VITT.

Anti-SARS-CoV-2 First Dose Vaccination in Previous 4–28 Days
Female gender with age less than 55 y
Unusual sites of venous thrombosis (e.g., jugular, cerebral sinuses or splanchnic veins)
Headache, fatigue, possible fever, abdominal discomfort and dyspnea
Occurrence of arterial thrombosis
Decrease in platelet count (e.g., 20% or more)
High D-dimer values with normal or decreased fibrinogen
Anti-PF4-Hep antibodies with ELISA testing

**Table 2:** Clinical conditions associated with immune thrombocytopenia and possible thrombosis.

Heparin-Induced Thrombocytopenia
Immune thrombocytopenia
Antiphospholipid syndrome
Evans syndrome
Erythematosus systemic lupus
Thrombotic thrombocytopenic purpura
Drug-induced thrombocytopenia
Bone marrow diseases (metastatic disease, myelodysplastic syndrome, aplastic anemia)
Recent transfusions
Infections (HIV, HBV, HCV)
Inherited thrombocytopenia: congenital amegakaryocytic thrombocytopenia, Wiskott-Aldrich syndrome, thrombocytopenia absent radius (TAR) syndrome, radioulnar synostosis, Bernard-Soulier syndrome, Von Willebrand disease.
